# Prevalence of inflammatory back pain and radiologic sacroiliitis is increased in patients with primary Sjögren’s syndrome

**DOI:** 10.11604/pamj.2018.30.98.15588

**Published:** 2018-06-05

**Authors:** Rafet Eren, Meryem Can, Fatma Alibaz-Öner, Sibel Yilmaz-Oner, Baris Yilmazer, Ayse Cefle, Haner Direskeneli, Pamir Atagündüz

**Affiliations:** 1 Department of Hematology, University of Health Sciences, Istanbul Training and Research Hospital, Istanbul, Turkey; 2 Department of Rheumatology, Medipol University, Faculty of Medicine, Istanbul, Turkey; 3 Department of Rheumatology, Marmara University, Faculty of Medicine, Istanbul, Turkey; 4 Department of Rheumatology, Bakirköy Sadi Konuk Training and Research Hospital, Istanbul,Turkey; 5 Department of Rheumatology, Kocaeli University, Faculty of Medicine, Kocaeli, Turkey

**Keywords:** Inflammatory back pain, axial spondyloarthritis, primary Sjögren´s Syndrome

## Abstract

**Introduction:**

The prevalence of Sjögren's syndrome (SS) in patients with the diagnosis of SpA has been reported to be higher than normal population. Yet, the vice-versa is unclear. In this study, we aimed to investigate the prevalence of IBP, radiologic sacroiliitis and SpA in patients with primary SS.

**Methods:**

85 patients followed at the rheumatology clinics of the Marmara and Kocaeli Universities with the diagnosis of primary SS between November 2011 and August 2012 were included in this study. The control group consisted of 100 age-and gender-matched patients. Inflammatory back pain and axial SpA were diagnosed according to the assessment of spondylo arthritis International Society (ASAS) criteria.

**Results:**

83 patients were (97%) female and 2 (3%) were male. Mean age of the patients was 49.1 (±11) years. Mean disease duration was 7.3 (±4) years. The patient and control groups were comparable in terms of age and gender (p > 0.05). Inflammatory back pain was observed in 21 (24.7%) of 85 primary SS patients and in 4 (4%) of 100 control subjects (p < 0.001), radiographic sacroiliitis was demonstrated in 9 (10.5%) of primary SS patients and 2 (2%) of the control subjects (p = 0.025). Remaining SpA findings were not encountered in either group.

**Conclusion:**

inflammatory back pain and radiologic sacroiliitis is increased in patients with primary SS. Whether IBP, SI joint inflammation and radiologic sacroiliitis is due to the co-existence of SpA and primary SS or IBP is an underdiagnosed clinical feature of SS deserves further studies of large patient numbers.

## Introduction

Sjögren's Syndrome (SS) is a systemic chronic inflammatory disease characterized by lymphocytic infiltration of exocrine glands [1]. Although dry eye and xerostomia due to the involvement of lacrimal and salivary glands are the major features of SS, other organ systems such as respiratory, urinary, neurological, gastrointestinal and hematological systems may be affected due to the involvement of related exocrine glands, as well [2-4]. Sjögren's syndrome is denoted as primary SS (pSS) when it exists solely and as secondary SS when it is associated with another autoimmune disease [5]. Rheumatoid arthritis (RA), systemic lupus erythematosus (SLE) and scleroderma are the most common autoimmune diseases associated with secondary SS [1-6]. Spondylarthropathies (SpA) comprise a group of inflammatory disorders, which are similar to each other in terms of clinical features and genetic susceptibility [7]. Ankylosing spondylitis (AS) is the prototype of these disorders and present particularly with inflammatory back pain (IBP) and radiologically-detected sacroiliitis [8]. The prevalence of SS in patients with the diagnosis of SpA has been reported to be higher compared to control groups and population surveys in previous studies [9-14]. Yet, the vice-versa is unclear. No sufficient data have been found about the frequency of IBP and SpA in primary SS patients. In this study, we aimed to investigate the prevalence of IBP, radiologic sacroiliitis and SpA in patients with a previous diagnosis of primary SS.

## Methods

Eighty five patients followed at the rheumatology outpatient clinics of the Marmara and Kocaeli Universities with the diagnosis of primary SS between November 2011 and August 2012 were included in this study. The primary SS diagnosis was done according to the revised version of the European criteria for SS proposed by the American-European Consensus Group [15]. Pregnancy and age under 18 were used as exclusion criteria. The control group consisted of 100 age- and gender-matched patients. Patients with any chronic inflammatory disease were excluded from the control group. Exclusion criteria for the patient group were applied to the control subjects, as well. We evaluated the primary SS patients and the control subjects using both clinical and laboratory parameters. Inflammatory back pain and axial SpA (axSpA) were diagnosed according to the Assessment of SpondyloArthritis International Society (ASAS) criteria [16]. Firstly, all pSS patients were questioned for the presence of chronic back pain with at least three months of duration at any time before the age of 45. Patients were questionned using the following criteria: 1) Onset before the age of 40 2) Insidious onset 3) Improvement with exercise 4) No improvement with rest 5) Pain at night (with improvement upon getting up). Subjects with four of these five features were defined as patients with IBP. Both patients and control subjects were screened for the presence of the following SpA findings, as well; IBP, arthritis, enthesitis, uveitis, dactylitis, psoriasis, inflammatory bowel disease, good response of back pain to nonsteriodal anti-inflammatory drugs and increased C-reactive protein levels. Subjects, who had at least one SpA finding, were screened for the presence of sacroiliitis with plain X-ray films and underwent further evaluation with computed tomography (CT). All X-ray films and CT scans were evaluated by the same radiologist from Marmara University, Department of Radiology. Bilateral grade 2-4 sacroiliitis or unilateral grade 3-4 sacroiliitis on X-ray films were considered as positive according to 1984 Modified New York Criteria [17]. Contour irregularities, erosions, subchondral sclerosis, spur formation, transarticular bone bridges and total ankylosis on CT imaging were considered as sacroiliitis findings [18]. Radiologist was ignorant of whether the images belonged to an AS patient or control subject. HLA-B27 status of the patients was obtained from the patient files. The study protocol has been approved by the local ethical committee on clinical research (Date: 25.02.2012 Number: B.30.2.MAR.0.01.02/AEK/195 Protocol: 09.2012.0023) and written informed consent was obtained from all patients. Statistical evaluation was performed with SPSS software (SPSS Inc., Chicago, IL, USA). Data were described as numbers and percentage or mean and standard deviation, when appropriate. x2 Fisher's exact test was used for evaluating categorical values and student-t test for continuous values in patient groups. All p-values were 2-sided with statistical significance at 0.05 alpha levels.

## Results

Eighty five primary SS patients and 100 control subjects were included in the study. The demographic characteristics of patient and control groups are presented in Table 1. Eighty three of the patients were (97%) female and 2 (3%) were male. Mean age of the patients was 49.1 (±11) years. Mean disease duration was 7.3 (±4) years. 98 (98%) individuals from the control group were female and 2 (2%) were male. Mean age of the control subjects was 49.3 (±12.2) years. The patient and control groups were comparable in terms of age and gender (p > 0.05) (Table 1). The disease characteristics of the patients are summarized in Table 2. All patients (100%) had dry eye and xerostomia with Schirmer's test positivity in 76 (89%) patients. Anti-Ro/anti La antibody positivity was present in 62 (72%) patients. Parotis scintigraphy was consistent with SS in 39 (45%) patients. Salivary gland biopsy confirmed the diagnosis of SS in 48 (56%) patients (Table 2).

**Table 1:** Demographic characteristics of the patient and control groups

	Primary SS	Control	P value
Age, mean (y) (±SD)	49,1 (±11)	49,3 (±12.2)	> 0.05
Gender (F/M) (%)	83/2 (%97-%3)	98/2 (%98-%2)	1
Disease duration, mean (y) (±SD)	7.3 (±4)	NA	

F: Female; M: male; SD: Standard deviation; SS: Sjögren’s syndrome; y: years

**Table 2:** Disease characteristics of the primary Sjögren’s syndrome patients

Patients	N= 85
Dry mouth	85 (100%)
Xerostomia	85 (100%)
Schirmer’s test positivity	76 (89%)
Anti Ro/La positivity	62 (72%)
Parotis scintigraphy consistent with SS	39 (45%)
Positive salivary gland biopsy	48 (56%)

**Prevalence of IBP and radiologic sacroiliitis**: Inflammatory back pain was observed in 21 (24.7%) of 85 primary SS patients and in 4 (4%) of 100 control subjects (p < 0.001) (Table 3). Among the SpA findings other than IBP, only increased serum CRP levels were found in 3 primary SS patients and these 3 patients were among the ones, who also had IBP. Fourteen of the 21 patients with inflammatory back pain were tested for HLA B27 and 2 were found to be HLA B27 positive. None of the 4 patients with inflammatory back pain in the control group had HLA B27 positivity. Remaining SpA findings were not encountered in either the patient or control groups. Sacroiliac imaging with plain X-ray and CT scans was performed in all 25 subjects with IBP (21 patients, 4 controls). The presence of radiographic sacroiliitis was confirmed by sacroiliac CT scans in 9 (42.8%) of 21 primary SS patients and 2 (50%) of 4 control subjects. Thus, radiographic sacroiliitis was demonstrated in 9 (10.5%) of primary SS patients and 2 (2%) of the control subjects (p = 0.025) (Table 3).

**Table 3:** Comparison of inflammatory back pain and sacroiliitis between the patient and control groups

	Primary SS	Control	P value
Inflammatory back pain	21/85 (%24.7)	4/100 (%4)	< 0.001
Sacroiliitis proven by X-ray/CT	9/85 (%10.5)	2/100 (%2)	P=0,025

CT: computed tomography; SS: Sjögren’s syndrome

## Discussion

Sjögren's Syndrome occurring in association with SpA and/or AS has been reported previously [9]. However, IBP is not among the expected findings of SS. Coexistence of IBP and SS in a patient was first described by Chang et al who assessed this condition as an abnormal finding [10]. Afterwards, Golstein et al described 2 cases having SS and AS concurrently for the first time. Similar to the former case, they denoted this situation as in favor of a chance association [9]. Beyond those mentioned cases, there is a lack of elaborative investigations about the existence of IBP and SpA in primary SS patients. The prevalence of IBP and SpA was firstly evaluated in primary SS patients in our study, where the prevalence of IBP was 24.7% in the patient group, and 4% in the control group. These findings were comparable with the results reported by Weisman et al who found the prevalence of IBP to be 5 to 6% in healthy subjects in a relatively large cohort (n = 5013) [19]. When we compare criteria defining IBP, ASAS criteria have higher specificity (91.7%) than Berlin and Calin criteria, yet it has low sensitivity (77%). None of the criteria for IBP is adequate in terms of both specificity and sensitivity, we preferred to use ASAS, which has the highest specificity [16,20,21]. Despite the discrepancies between the underlying pathophysiological mechanisms, the association of SS with psoriatic arthritis, AS and reactive arthritis was reported by Whaley et al at 1971 [11]. Later, Di Fazone et al showed that SS European criteria was met more often in SpA patients compared to the control subjects (31% vs. 2.9%) in a study including 41 SpA patients and 102 control subjects [12]. However, Brandt et al found a lower prevalence of SS in SpA patients (7.6%) in a higher number of patient (n = 105) and control (n = 150) groups [13]. A similar result was showed by Gusis et al in a relatively low number of SpA patients (n = 22), where only 2(9,6%) patients had SS [14]. In the above-mentioned studies investigating the presence of SS in SpA and/or AS patients, the timing of SS-whether it started prior to or after the onset of SpA-has not been conceived clearly. In contrast to the previous studies, we investigated the prevalence of inflammatory back pain, radiologic presence of sacroiliitis and SpA in primary SS patients and found a higher prevalence (24.7%) for IBP and (10.5%) radiologic sacroiliitis. In addition, in our study, the presence of IBP, radiologic sacroiliitis and axial SpA were investigated in primary SS patients who have been followed for a relatively longer period of time.

Sacroiliitis findings on radiological imaging develop nearly 5 to 10 years after the onset of IBP in almost half of the patients [22]. Therefore, although direct radiography assessed using modified New York criteria remains the initial imaging modality for detection of sacroilitis in the ASAS criteria, magnetic resonance imaging may be needed as second-line method of choice when modified New York criteria are not met [16]. Nine patients and two controls with IBP had radiographic sacroiliitis in our study. We therefore did choose to proceed with SI joint CTs instead of MRIs since an MRI is not obligated according to ASAS axial SpA criteria and SI joint CT is superior in detecting chronic bony changes. Due to the oblique course and corrugated edges of the sacroiliac joint and also the superposition of the surrounding tissues, joint distance cannot be evaluated correctly with conventional radiography. Computed tomography, by demonstrating joint distance narrowing, subchondral sclerosis, erosions and ankylosis, has taken an important role in evaluation of sacroiliitis [23-26]. Carrera et al showed the superiority of CT scanning compared to direct X-ray in a study evaluating sacroiliitis in 17 symptomatic patients. While sacroiliitis was detected in 12 patients with CT, only 5 of these patients showed sacroiliitis on X-ray [26]. Similar results were found by Kozin et al in a study including 43 patients who had sacroiliitis clinically. 56% of the patients had sacroiliitis on CT scans; yet only 21% of the patients had suspicious findings on X-ray [24]. Beside these studies, Puhakka et al showed that both CT and MRI are equally sufficient in detection of sacriliitis [27]. Morover, CT has been found to be more useful and cheaper in demonstration of bone erosions, sclerosis and ankylosis in some studies [28-30]. Based on our previous clinical observation of a possibly increased IBP prevalence in primary SS patients, we aimed to investigate a possible increase of axial SpA in primary SS patients. Our results imply that there is an actual increase in the prevelance of IBP. But interestingly, patients with IBP and CT-proven sacroiliitis had no additional SpA features. Clearly, a definition of axial SpA in primary SS patients based solely on IBP and radiologic sacroiliitis is not adequate. Although these patients would fulfill the classification criteria of ASAS for axial SpA, one should keep in mind that the presence of other frequent and specific criteria for SpA increases the possibility of a SpA diagnosis. In our minds, the critical observation here is the increased frequency of IBP and sacroiliitis in primary SS patients. Whether IBP, SI joint inflammation and radiologic development of sacroiliitis is due to the co-existence of SpA and primary SS or IBP is an under-diagnosed clinical feature of SS deserves further studies of large patient numbers. One weakness of our study may be that it leaves the question of, whether MRI should be a part of the laboratory investigations in primary SS patients with IBP open. Another weakness was that a part of our data collection depended on patients' past remembrance and therefore its accuracy was limited with patient memory.

## Conclusion

In conclusion, inflammatory back pain and radiologic sacroiliitis is increased in patients with primary SS. Whether IBP, SI joint inflammation and radiologic sacroiliitis is due to the co-existence of SpA and primary SS or IBP is an underdiagnosed clinical feature of SS deserves further studies of large patient numbers.

### What is known about this topic

- The prevalence of Sjögren's syndrome in patients with the diagnosis of SpA is higher than normal population;
- There is no sufficient data on the frequency of IBP and SpA in primary SS patients.

### What this study adds

- Inflammatory back pain and radiologic sacroiliitis is increased in patients with primary SS;
- IBP, SI joint inflammation and radiologic sacroiliitis is either due to the co-existence of SpA and primary SS or IBP is an underdiagnosed clinical feature of SS.

## Competing interests

The authors declare no competing interests.
